# Cross-validation of predictive models for functional recovery after post-stroke rehabilitation

**DOI:** 10.1186/s12984-022-01075-7

**Published:** 2022-09-07

**Authors:** Silvia Campagnini, Piergiuseppe Liuzzi, Andrea Mannini, Benedetta Basagni, Claudio Macchi, Maria Chiara Carrozza, Francesca Cecchi

**Affiliations:** 1 The Biorobotics Institute, Scuola Superiore Sant’Anna, Viale Rinaldo Piaggio 34, 56025 Pontedera, Italy; 2 IRCCS Fondazione Don Carlo Gnocchi Onlus, Via di Scandicci 269, 50143 Florence, Italy; 3 Department of Experimental and Clinical Medicine, University of Florence, Largo Brambilla 3, 50134 Florence, Italy

**Keywords:** Predictive models, Prognosis, Stroke, Machine learning, Rehabilitation

## Abstract

**Background:**

Rehabilitation treatments and services are essential for the recovery of post-stroke patients’ functions; however, the increasing number of available therapies and the lack of consensus among outcome measures compromises the possibility to determine an appropriate level of evidence. Machine learning techniques for prognostic applications offer accurate and interpretable predictions, supporting the clinical decision for personalised treatment. The aim of this study is to develop and cross-validate predictive models for the functional prognosis of patients, highlighting the contributions of each predictor.

**Methods:**

A dataset of 278 post-stroke patients was used for the prediction of the class transition, obtained from the modified Barthel Index. Four classification algorithms were cross-validated and compared. On the best performing model on the validation set, an analysis of predictors contribution was conducted.

**Results:**

The Random Forest obtained the best overall results on the accuracy (76.2%), balanced accuracy (74.3%), sensitivity (0.80), and specificity (0.68). The combination of all the classification results on the test set, by weighted voting, reached 80.2% accuracy. The predictors analysis applied on the Support Vector Machine, showed that a good trunk control and communication level, and the absence of bedsores retain the major contribution in the prediction of a good functional outcome.

**Conclusions:**

Despite a more comprehensive assessment of the patients is needed, this work paves the way for the implementation of solutions for clinical decision support in the rehabilitation of post-stroke patients. Indeed, offering good prognostic accuracies for class transition and patient-wise view of the predictors contributions, it might help in a personalised optimisation of the patients’ rehabilitation path.

## Introduction

The World Health Organization defined stroke as: “rapidly developing clinical signs of focal (or global) disturbance of cerebral function, with symptoms lasting 24 h or longer or leading to death, with no apparent cause other than of vascular origin” [1]. In fact, stroke is the second leading cause of death worldwide [2] and despite the advances in healthcare contributing to the reduction of the mortality rate, millions of people have to deal with physical and/or psychological burdens affecting their quality of life [3, 4].

Rehabilitation treatments and services are the key elements for the recovery of patients’ functions, independence, and quality of life [5]. However, given the increasing number of available therapies for rehabilitation, a lack of consensus among measures compromises the possibility to fully optimise the clinical outcomes and to determine an appropriate level of evidence for treatments. For this reason, an accurate and comprehensive assessment is essential, to deeply analyse the factors influencing patients’ recovery and support the clinical decision for personalised treatment.

The growing tendency toward evidence-based medicine and data-driven rehabilitation promoted further interest in Clinical Decision Support Systems (CDSS) [6], showing among their functions and advantages the possibility to contain costs, bolster clinical workflow and efficacy, favour patients’ safety, support diagnosis, and promote treatment paths customisation. Within CDSS, knowledge-based and non-knowledge based systems can be distinguished, differentiating respectively in the use of evidence-based rules (determined on clinical experience or literature or patient-directed indications) or Artificial Intelligence (AI) algorithms. Despite the controversial aspects related to the reliability and safety of these systems, particularly important in clinical applications, the use of AI and Machine Learning (ML) for Intelligent Decision Support Systems is being widely explored [7].

For what concerns ML applications in post-stroke rehabilitation, research is still in a development phase, with extensively large numbers of studies evaluating longitudinal associations among features and discharge or long-term outcomes [8], and more limited studies dedicated to the development and validation of predictive models [9, 10, 11]. However, cross-validated ML models for prognosis of functional level on stroke cohorts are indeed generating a growing interest [12]. The analysis of the literature reveals a great heterogeneity both in the selection of predictors, often limited to the available scales in use in the setting, and outcome measures [12].

One of the most recurrently addressed functional outcomes is the Barthel Index (BI) scale [13], the gold standard tool for functional independence and basic daily living activities in the stroke population [14]. Among some examples in the literature, Sale et al. [15] worked on 3 Support Vector Machine (SVM) models with nested cross-validation on a cohort of 55 sub-acute post-stroke patients, aiming at a prediction of the BI score at discharge. The results indicated the great importance of the patients’ inflammatory and clinical descriptors at baseline and that the specific stroke aetiology does not significantly influence the results on the prediction (correlation coefficient: 0.75, Root Mean Square Error: 22.6, Mean Absolute Deviation Percentage: 84.0%). Lin et al. [16] obtained 0.72 (0.04) and 0.68 (0.03) on the average (standard error) sensitivity and specificity respectively on a cross-validated SVM model predicting a three-classed discretised BI score.

We are fully convinced that, for a reliable application of CDSS, the assessment of the generalisability of the proposed results is crucial. Such assessment, achieved by the implementation of proper validation approaches within the models, allows estimating how the solution will be accurate when processing new data. The same analysis of the literature reveals a limited use of external [17, 18, 19] or nested cross-validation approaches [15, 20], toward a more diffuse use of split-sample, bootstrap or non-repeated cross-validation methods. For this reason, the current study attempts to extend and generalise the results obtained by classical statistical analysis employing nested cross-validated ML algorithms, with a specific focus on the models' interpretability.

Finally, as previously mentioned, a commonly raised issue concerning the ethical use of ML is the lack of interpretability. Classical solutions are already known to provide model-based feature importance (e.g. regressions coefficients, Gini index in tree-based models, etc.…). Nevertheless, such rankings are built on the full dataset and are not patient-specific. In our work, we propose the use of Shapley values, via the Shapley Additive exPlanations (SHAP, [21]) technique, allowing us to provide clinicians with a patient-wise explanation of the prediction. A patient-specific interpretation analysis, explaining how single factors contribute to the outcome estimate for individual patients, would improve the quality of interaction with the clinical team. Providing details on the predictors contributions in the outcome estimation for each patient can make the data-driven solution worth of the clinicians’ trust [22, 23].

In synthesis, our aim is to cross-validate on a retrospective database an interpretable model targeting the modified Barthel Index at discharge after intensive post-stroke rehabilitation, analysing the contribution of each prognostic factor to the prediction through the use of the SHAP technique. This last analysis will give us the possibility to confirm the results obtained through statistics.

## Methods

### Sample

This study was based on data collected retrospectively on a cohort of 278 post-stroke patients admitted at two Intensive Rehabilitation Units (IRUs) of Fondazione Don Carlo Gnocchi (S. Maria alla Pineta, Massa, and S. Antonio Abate Hospital, Fivizzano) between January 2015 and August 2017. The inclusion criteria considered were the following:

- Diagnosis of ischemic or haemorrhagic stroke
- Age over 18 years
- Hospitalisation period between January 2015 and August 2017

The study was conducted following the Helsinki Declaration and it was approved by the local ethical committee (Comitato Etico Regionale per la Sperimentazione Clinica della Toscana Area Vasta Nord-Ovest, Prot. N. 18178, 10/09/2020). The informed consent for the use of data was collected afterwards.

Each patient received at least three hours of rehabilitation per day. All patients underwent clinical observation, nurse management, and physiotherapy. The physiatrist prescribed speech/deglutologic training, neuropsychological treatment, and occupational therapy, when needed, and psychological support was available for both patients and caregivers.

Data were collected at admission and discharge of intensive rehabilitation treatment, and the collected variables used in this study concerned demographics and clinical, functional and cognitive evaluations.

The Individual Rehabilitation Project, which included a standard assessment protocol, was defined according to the International Classification of Functioning, Disability and Health (ICF) [24], the SPREAD 2011 guidelines [25], and the IPER2 model [26].

### Outcome

**Fig. 1:**
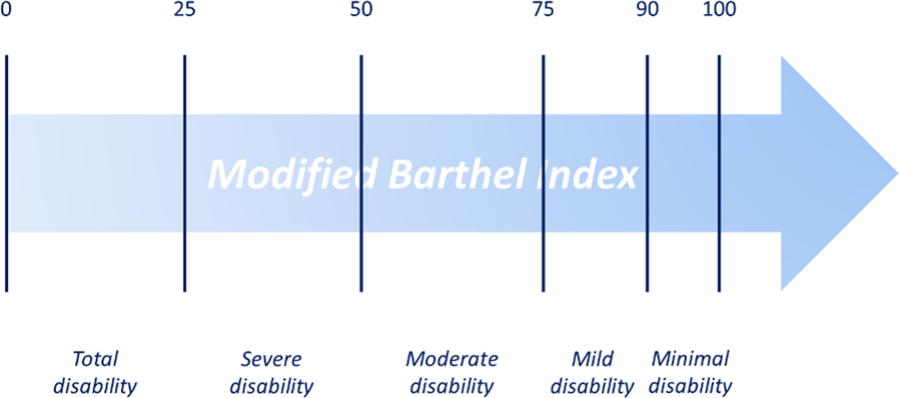
Modified Barthel Index cut-off values and the associated disability levels

The functional recovery was the outcome considered in this work and it was measured as the class transition on the Modified Barthel Index (mBI) scale. More in detail, a categorisation of the mBI in six classes was performed both on the admission and discharge scale with the following cut-off values: 0–24, 25–49, 50–74, 75–89, 90–99, 100 [27, 28]. In this study, for numerosity reasons, classes corresponding to cut-offs of 90–99 and 100 were collapsed, obtaining 5 different groups representing, in ascending order, total, severe, moderate, mild and minimal disability levels (Fig. 1). The dichotomous outcome of class transition was obtained giving value 1 when the patients were experiencing a class improvement from admission to discharge.

Patients with admission mBI scores corresponding to the highest class were removed from the analysis, given their impossibility to transition toward a higher class that would necessarily lead to the attribution of a value of 0 in the outcome.

### Predictors

For what concerns the independent variables, i.e. the candidate predictors for the selected outcome, the following features collected at admission were selected:

- Age
- Gender
- Bladder catheter presence
- Bedsores presence
- Stroke aetiology
- Comorbidity, assessed by the Cumulative Illness Rating Scale, CIRS (total score) [29]
- Disability in communication, independent from the cause (aphasia, dementia, deafness etc.…), assessed by Communication Disability Scale, SDC [30]
- Premorbid disability, assessed by the anamnestic Modified Rankin Scale, mRS [31]
- Deambulation, assessed by the Standardised Audit of Hip Fracture in Europe, SAHFE (total score) [32]
- Disability in Activities of Daily living, assessed by the Modified Barthel Index, mBI (total score) [13]
- Trunk control, measured by the Pain Trunk Control Test, TCT (total score categorised in four ordinal groups based on the 25, 50 and 75 percentiles) [33]
- Pain, measured by a 10 points Numerical Rating Scale, NRS [34]
- Cognitive status, assessed by the Mini-Mental State Examination, MMSE (adjusted total score) [35]

All the listed features entered the model and underwent descriptive analyses employing mean and standard deviation, or median and interquartile range when appropriate, for numerical variables and relative frequencies for the categorical ones. Missing values on the features were treated via statistical imputation with median values or mode values for numerical or categorical variables, respectively. The statistical imputation method was applied only to those variables for which the completeness percentage was at least 70%. Variables completed in less than 70% of the records were discarded due to exceeding number of missing values.

All the descriptive analyses of the features were performed on IBM Corp. Released 2020. IBM SPSS Statistics for Windows, Version 27.0. Armonk, NY: IBM Corp.

### Model implementation

ML methods were implemented in Python, specifically using *Scikit-learn* library [36]. After features normalisation, four different machine learning algorithms [Elastic-Net regularized logistic regression, Support Vector Machine (SVM), Random Forest (RF), and k-Nearest Neighbours (kNN)] were trained, optimising on accuracy values, and validated with nested cross-validation. An inner fivefold cross-validation loop was deployed for hyper-parameters optimization, while an external tenfold loop was adopted for test set identification. Given the unbalanced distribution of the outcome classes, the models were optimised using the *balanced accuracy* (BA) metric. For what concerns the models optimisation, in Table 1 reports the list of the parameters ranges over which each algorithm was optimised and it was done with the Optuna library.

**Table 1:** Description and ranges of optimisation of the parameters for each algorithm trained

Classifier	*Parameters* (description)	Values range
*Logistic Regression*	*c* (inverse of the regularisation strength)	0.001–1000
	*l1_ratio* (to select the weight of L1 and L2 penalties)	0.1–0.9
*kNN*	*n_neighbors* (to select the number of neighbours)	10–50
	*weight* (to select a uniform or distance-based weight on the samples)	“uniform”, “distance”
	*algorithm* (to select the type of algorithm to compute the nearest neighbours)	“brute”, “ball-tree”, “kd_tree”
	*leaf_size* (parameter selectable only for tree-based algorithms that affect its speed and memory)	5–100
	*p* (power of the Minkowski metric for the distance calculation)	1–5
*SVM*	*gamma* (kernel coefficient)	10^–6^–10^6^
	*C* (inverse of the regularisation strength)	10^–6^–10^6^
	*kernel* (kernel type to be used in the algorithm)	“rbf”, “linear”
*RF*	*n_estimators* (number of trees in the forest)	5–25
	*max_depth* (maximum depth of the tree)	1–10
	*max_features* (to select the number of features to consider when looking for the best split)	2–10
	*criterion* (to select the function type to estimate the quality of the split)	“gini”, “entropy”
	*min_samples_leaf* (to select the minimum number of samples to have a leaf node)	3–10
	*min_samples_split* (to select the minimum number of samples to split and internal node)	5–20
	*bootstrap* (to activate or not the bootstrap approach when building the trees)	“true”, “false”

The performances of the different models were compared in terms of accuracy and BA, obtained with the following calculation:

$$BA=\frac{\frac{TP}{(TP+FN)}+\frac{TN}{(TN+FP)}}{2}$$

with: TP: True positive values in the confusion matrix; TN: True negative values in the confusion matrix; FP: False positive values in the confusion matrix; FN: False negative values in the confusion matrix.

Moreover, their predictions were further combined in two different ways, obtaining two additional results. First, predictions of all models were concatenated and the mode of the predictions vector was considered as the final prediction (*majority voting).* Then, predictions posterior probabilities of both classes were summed and the class with the highest cumulative posterior probability was chosen as the final prediction (*weighted posterior voting*) [37].

Further, on the best performing solution, chosen on inner loop results, the *SHAP* method was used for interpretability analyses aiming at understanding the effects of the features on the outcome prediction on test-set patients. More specifically, the interpretability of the model is achieved through the calculation of the Shapley values from game theory, allowing for a better understanding of the contribution of each feature entering the model. Thus, given a specific feature instance, its Shapley value will be calculated as the average value, among all possible feature coalitions, of the difference between the prediction of the model with and without the specific instance.

## Results

From a total number of 278 patients, a final sample of 273 patients was obtained after the removal of patients with the highest mBI class at admission (the median and IQR of the mBI scores of the removed patients was 94 [IQR = 8]). In the selected sample, the median length of stay was of 40 days (IQR = 15). A total of 181 (66.3%) patients experienced class transition, however, among the remaining 92 (33.7%), 66 patients had an improvement on the continuous mBI value, 25 patients remained stable, and only 1 patient underwent a decrease in the mBI scale. All the identified variables were included in the analyses, as the percentage of missing values never exceed the 30% threshold, and the missing values present were filled with median imputation. The descriptive analyses of the features fed into the model are shown in Table 2.

**Table 2:** Descriptive analyses of the sample, concerning the independent variables, collected at admission, and the outcome (class transition), collected at discharge

Variables		Descriptives Mean (std)/Median [IQR] or frequencies
Predictors (collected at admission)		
Categorical features	Gender (0: Male; 1: Female)	0: 134; 1:144
	Bladder catheter (0: Absent; 1: Present)	0: 179; 1: 99
	Pressure ulcers (0: Absent; 1: Present)	0: 233; 1: 45
	Stroke aetiology (1: Ischemic; 2: Haemorrhagic; 3: Both)	1: 208; 2: 55; 3: 15
Numerical features	Age	79 [IQR = 14]
	CIRS	22 [IQR = 6]
	SDC	2.72 (1.35)
	mRS (premorbid)	1 [IQR = 2]
	SAHFE	5 [IQR = 0]
	mBI	16 [IQR = 41]
	TCT	36 [IQR = 87]
	NRS	0.91 (2.23)
	MMSE	22.00 [IQR = 8.25]
Outcome (collected at discharge)		
	Class transition (0: No transition; 1: Transition)	0: 186; 1: 92
	mBI	57 [IQR = 62]

Predictors are presented according to the type of variables; numerical and categorical

*CIRS* Cumulative Illness Rating Scale, *SDC* Communication Disability Scale, *mRS* modified Rankin Scale, *SAHFE* Standardised Audit of Hip Fracture in Europe, *mBI* modified Barthel Index, *TCT* trunk control test, *NRS* Numerical Rating Scale, *MMSE* Mini-Mental State Examination

**Fig. 2:**
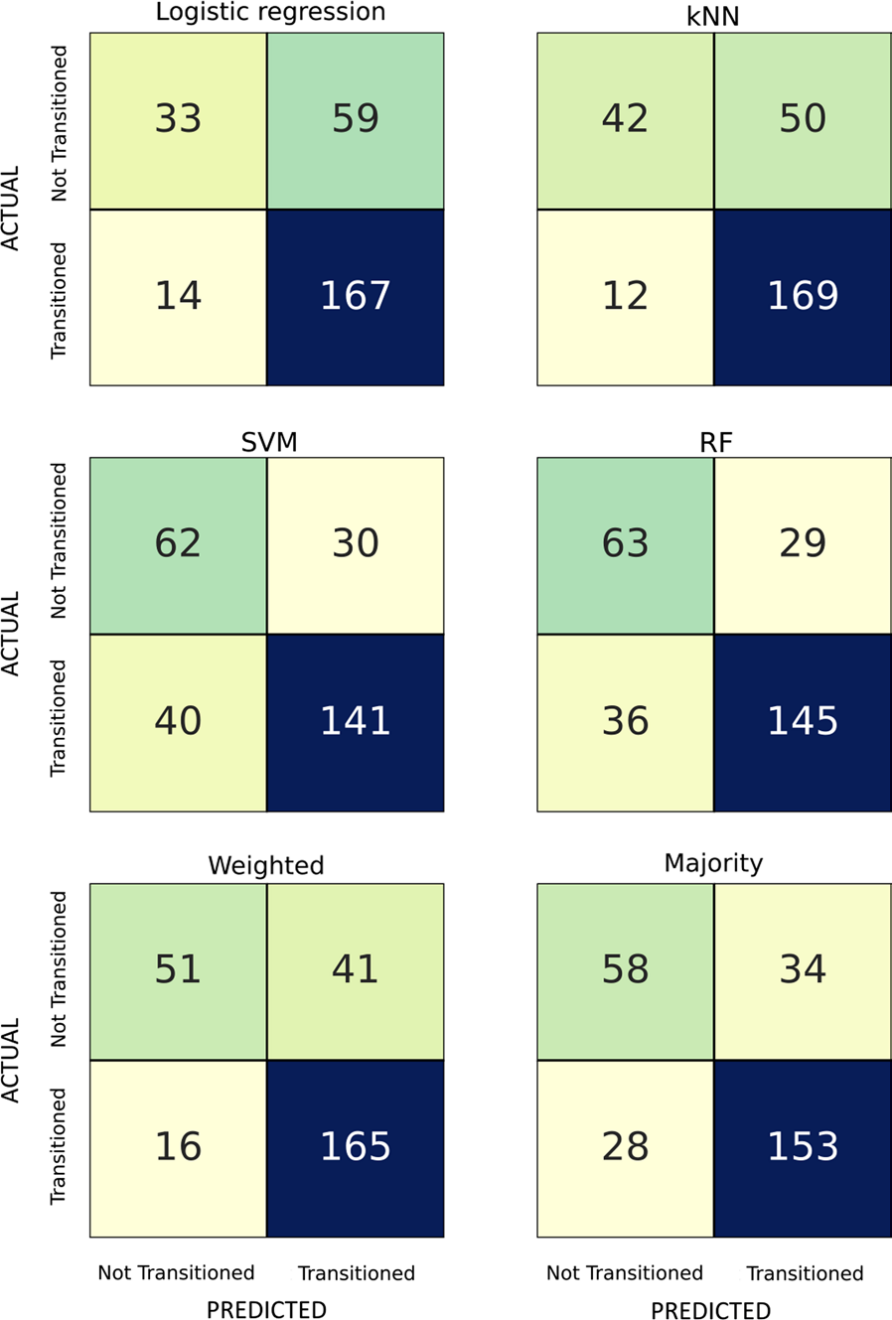
Confusion matrix of the different classifiers. For each algorithm, the actual and predicted values are presented in rows and columns, respectively

For all the classifiers a test set accuracy close to 75% on the class transition was obtained. Specifically, the kNN, SVM, RF and logistic regression obtained accuracies of 77.3%, 74.4%, 76.2%, and 73.3%, respectively. Additionally, the majority voting solution obtained 77.2% accuracy and the weighted one achieved the highest value with 79.1% (Fig. 2). For what concerns the performances in terms of BA, the kNN, SVM, RF and logistic regression obtained BAs of 69.5%, 72.6%, 74.3%, and 64.1%, respectively. The weighted and majority voting solutions reached 73.3% and 73.8%, respectively. Thus, when looking at the BA results, the RF algorithm performed better on the test set.

**Fig. 3:**
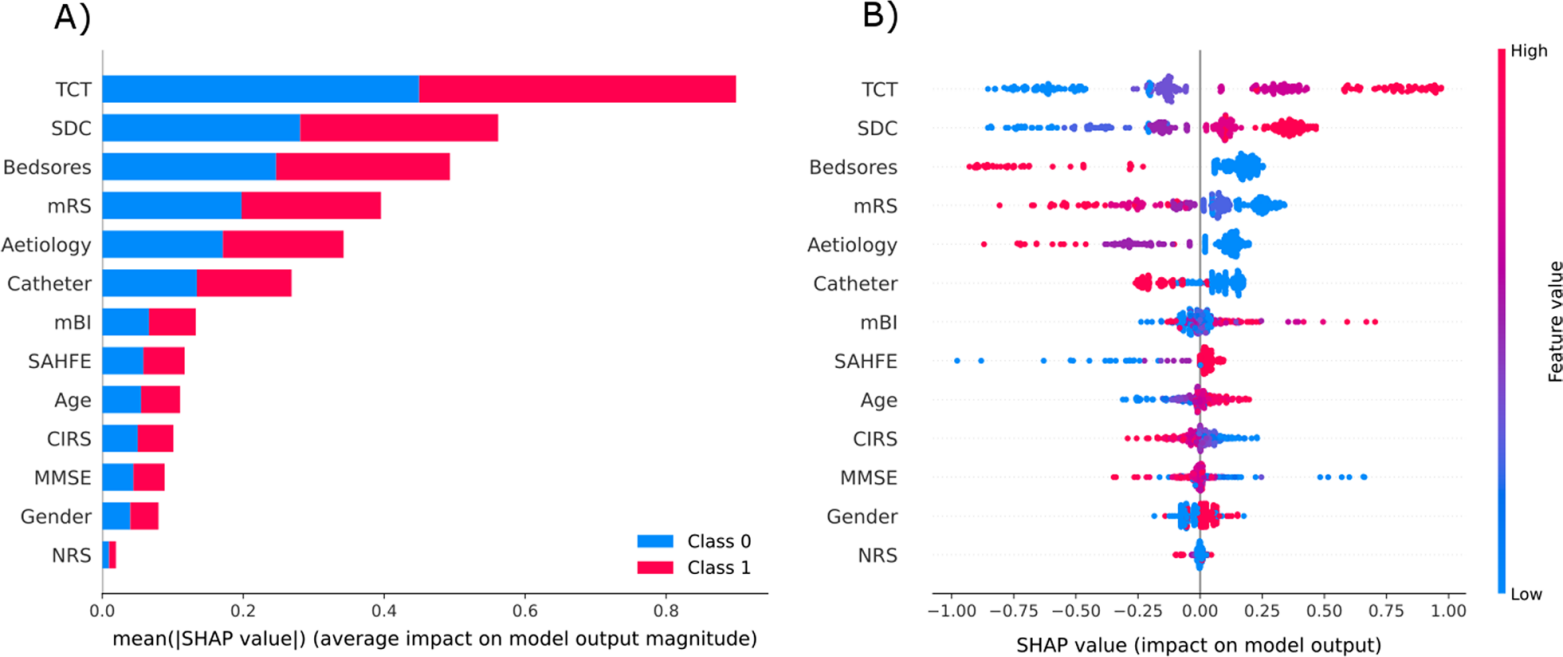
Contributions of the predictors entering the model with best validation accuracy (SVM classifier) on the class transition. In panel **A**, a bar plot of the contributions of each predictor to the two classes (in blue and red) is shown. In panel **B**, a beeswarm plot showing the Shapley values for each patient and feature-wise is presented. The colour of the dots is indicating how the sign of the feature is contributing to the prediction

Interpretability analysis was carried out on the best performing solution on the validation set (SVM, validation BA = 80.1%). The obtained results showed that higher values of TCT, bedsores absence, higher SDC values, mild disability level on the mRS, presence of ischemic stroke, and absence of bladder catheter strongly and positively impacted the class transition (Fig. 3A). Additionally, the patient-wise contribution of each feature is presented in Fig. 3B.

**Fig. 4:**
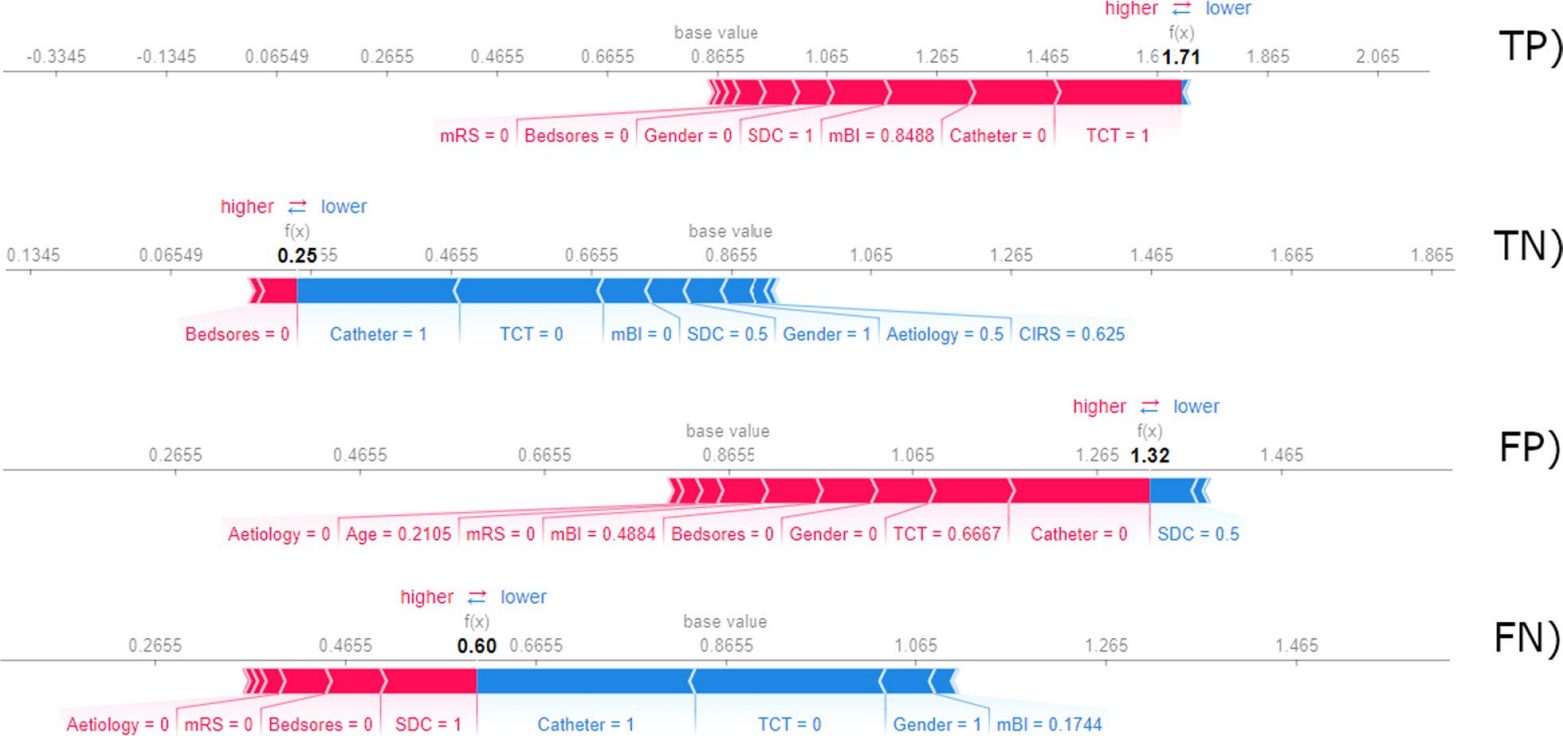
Examples of features contributions (normalised values) to the prediction for a patient correctly classified as transitioning (panel TP) and one as non-transitioning (panel TN) and a misclassified patient as transitioning (panel FP) and non-transitioning (panel FN). *Details:* Patient TP presented the following characteristics: Male, 62 years old, ischemic stroke, absence of catheter, absence of bedsores, CIRS = 27, mRS = 0, SDC = 4, TCT = 100, NRS = 4, MMSE = 18, SAHFE = 2, mBI at admission = 73, mBI at discharge = 94. Patient TN presented the following characteristics: Female, 87 years old, haemorrhagic stroke, presence of catheter, absence of bedsores, CIRS = 29, mRS = 1, SDC = 2, TCT = 0, NRS = 0, MMSE = 22, SAHFE = 5, mBI at admission = 0, mBI at discharge = 6. Patient FP presented the following characteristics: Male, 55 years old, ischemic stroke, absence of catheter, absence of bedsores, CIRS = 20, mRS = 0, SDC = 2, TCT = 61, NRS = 0, MMSE = 11, SAHFE = 4, mBI at admission = 42, mBI at discharge = 42. Patient FN presented the following characteristics: Female, 74 years old, ischemic stroke, presence of catheter, absence of bedsores, CIRS = 20, mRS = 0, SDC = 42, TCT = 0, NRS = 0, MMSE = 28, SAHFE = 5, mBI at admission = 15, mBI at discharge = 87

In Fig. 4, the contributions of each feature are represented for specific patients belonging to the test set. Both cases are predicted by the model as non-transitioning on the outcome; however, the first patient, on the top (panel A), is an example of the correctly classified outcome, the bottom one (panel B) is an example of misclassified. More specifically, on the abscissa of the graph, are represented the Shapley values and in bold the overall contribution of the features. In both cases, the overall contribution is lower than the “base value”, indicating the prediction toward a non-transition of the mBI class. The normalised values of the main contributing features are also presented.

## Discussion

The need for the application of more generalizable and robust methods for outcomes prediction in the post-stroke population has been advocated [8, 12, 38], in order to foster the implementation of CDSS within clinical routine. This aspect could have great importance in the rehabilitation context, both to improve patients’ outcomes and to contain costs of care. About this point, on a cohort of 1197 stroke patients, it has been demonstrated that the length of stay in the rehabilitation setting, accounting for the 70% of the total stroke costs, is strongly associated with the initial stroke severity and the improvement in the recovery [39]. Thus, promoting the optimisation of the rehabilitation path, and improving the clinical recovery is the key target for data-driven solutions. In order to reach a full implementation of data-driven based clinical decision support tools, it is crucial to develop robust and interpretable predictive models.

In this process, functional outcomes, such as the Modified Barthel Index scale, are a good first-step target, as they allow a comprehensive and higher-level evaluation of the patients’ independence [24]. In absence of a validated Minimal Clinical Important Difference on the mBI scale, the mBI in this study was dichotomised and considered as class transition, to determine a more clinically relevant functional recovery [28]. All the implemented classifiers obtained good accuracies, and the weighted results obtained through the sum of posterior probabilities obtained the highest accuracy (79.1%) (Fig. 3).

To compare the results obtained by Lin et al. [16] on the mBI categorised in three classes, sensitivity and specificity for the best classifier (RF) were obtained from the aggregated predictions performed on the test set folds. The results showed higher values both in terms of specificity (0.68 in the previous study and 0.68 in our case) and sensitivity (0.72 and 0.80 in our case). However, the comparison of these numbers should be done in light of the technical differences of the implemented solutions, specifically concerning the different outcome types and validation approaches. In fact, in our work, concerning the validation of the model, nested cross-validation was implemented for each classifier, similarly to what Sale et al. [15] proposed, ensuring a more robust analysis of results generalisability. Indeed, it has been shown that exclusively relying on cross-validation accuracy for both model selection, hyper-parameter tuning and evaluation of the results can carry a significant bias on the prediction (the so-called cross-validation bias [40]). Thus, an approach as nested cross-validation can ensure a reduction of the cross-validation bias, replicating error estimations similar to those obtained with independent external validation [40, 41, 42].

In addition to the development and nested cross-validation of the classifiers, an analysis of the interpretability of the best performing model was also performed. More specifically, the analysis of interpretability is conducted through the application of game-theory approaches that evaluate the weight of each feature on the prediction in a patient-specific manner [43]. Up to our knowledge, the only paper addressing these methods on stroke predictive models is the paper from Qin et al. [44], using prognostic models for the prediction of mortality. This technique gives an insight into the roles and mutual interactions among features and fosters the translational applicability of ML models in the clinical context. Indeed, the understanding of which aspects contribute to the given outcome prediction can empower the clinical users of the information on when such solutions can be trustworthy. Especially in the case of the misclassified patients, the variables obtained from the patients’ assessments, together with the factors contributing to the prediction in the model, can make the clinician understand and further analyse these cases. Moreover, enhancing the concept of personalised treatment optimisation, the interpretability through the use of Shapley values allows for patient-specific analyses of features contributions. As an example, Fig. 4 is representing the specific feature contributions for two patients of the test-set, classified as non-transitioning, specifically showing a correct classification (panel A) and misclassification (panel B) of the model. In panel B, it is visible how the clinical complexity of the patient, i.e. the presence of the bedsore, a global disability and the presence of the bladder catheter, is contributing in the decision of the model toward a non-transition on the mBI class. This information can be crucial for the clinicians in order to select the proper rehabilitation plan for the specific patient.

The analysis of the weights of factors with the *SHAP* method showed great importance on functional aspects such as the trunk control, communication level, disability level, bladder catheter and the pressure ulcers, rather than the mBI level at admission (Fig. 3). Additionally, the type of stroke, among ischemic, haemorrhagic or both, was confirmed as a predictor. Specifically, the presence of haemorrhage, either alone or in combination with ischemic stroke type, resulted in a worse outcome, representing a proxy of stroke severity at the entrance. This hypothesis was indeed confirmed by a statistically significant difference in mBI total score at admission between the two groups, with lower values for those experiencing haemorrhage (Mann-Whitney test, p-value = 0.007).

The results on trunk control are in line with the literature, showing that trunk control is an essential predictor of functional outcomes and activities of daily living [16, 45]. In fact, trunk control has a deep connection both with mobilisation tasks and the use of extremities. Trunk control is not only representing the ability to keep balance during the sitting and upright position but the capability to perform stabilisation and selectively control the movements of both the upper and lower trunk [46]. It is well known that the proximal stabilisation of the trunk is related to higher control of distal extremities and efficient walking is guaranteed by a proper rotation of the shoulders with respect to the pelvis. Also, Lin et al. [16], specifically developing predictive models on a three-classes mBI, obtained the trunk control as one of the key features involved in recovery.

Also related to mobilisation, the presence of markers of clinical complexity, such as bedsores or bladder catheter, was reported among the most significant predictors in the model. Especially in the first year post-stroke, immobility-related complications can be very common and negatively influence the functional outcome and the independence in basic activities of daily living. A study from Sackley et al. [47], on a cohort of 122 patients, reported 22% of patients suffered from bedsores within 12 months of observation. The same study additionally reported through preliminary analyses how the number of complications is negatively correlated with the Barthel Index score at three months post-stroke.

Finally, the communication level was another important aspect emerging from our results. In this work, the disability on the communication level was measured with the SDC scale. Despite the mBI scale does not directly measure communication components, it is noticeable the importance of communication levels on functional recovery. In the literature, the role of communication limitations, such as aphasia, is controversial [48, 49]. Like other measures of disability, SDC does not explain the specifics of the disorders (aphasia, apraxia, dysarthria, dementia, deafness) which, individually or in combination, can impair communication. The SDC evaluates the difficulties in communication as assessed by the clinician after an anamnestic interview and clinical examination. It may be affected by a combination of neurological problems, being this way an indicator for an aggregate of problems and a severity index. Hence, a disability in communication is necessarily associated with a reduced comprehension of therapeutic instructions and may prevent the development of the therapeutic relationship between the patient and the rehabilitation team, possibly delaying or compromising recovery [49]. Interestingly, in our study, the beeswarm plot (Fig. 3) is showing how the levels from 0 to 2, connected to total to moderate limitations in communicating, are predictive of an absence in class transition, whilst on the contrary levels 3 and 4 (mild and absent communication limitations) have strong positive predictive value on the class transition, as already reported for severe brain injuries [50].

Despite the retrospective nature of the study, the proposed ML methodology was validated through a nested cross-validation approach, ensuring high-level confidence of the achieved results in terms of generalisation capability. The results obtained were promising and could contribute to first-step evidence for the realisation of interpretable CDSS. As already suggested for different conditions, addressing explanation techniques for the output of intensive post-acute rehabilitation [26, 51] provided a data-driven focus on the importance of trunk control, bedsores and communication levels in the recovery of functional outcome of post-stroke patients at discharge from intensive rehabilitation. These aspects, which are in strong agreement with clinical evidence and practice [26, 51], further fostered the reliability and trustworthiness of the predictive model developed.

### Limitations and implications for future research

Despite further strategies could be investigated from the technical point of view, (e.g. oversampling techniques), the selection of the variables should be mostly discussed and possibly improved in future research. Indeed, the retrospective nature of the study implied the use of a restricted selection of variables related to limited aspects of the patients’ rehabilitation. For this reason, a prospective observational design was developed for a multifactorial analysis of post-stroke patients’ characteristics [52] and their role for the prediction of functional recovery.

Additionally, the selection of the outcome measure deserves some additional comments. As it was stated within the introduction, the development of predictive models is the first step in the direction of tools for the clinical decision support. Thus, as a preliminary stage, we decided to address to a more generic outcome that could broadly quantify the functional outcome of the patient at discharge. For this reason, we selected the class transition on the Modified Barthel Index, over other measures such as the discharge score, the difference between discharge and admission scores, efficiency, or effectiveness [53], due to its easier interpretation. We are aware some limitations may affect this choice, such as the fact that a linear relationship between the score and the clinical conditions of the patients is assumed, or that even a small change in the total score could lead to a transition, or the fact that transitions of one or more classes are equally considered. However, class transition was chosen since it provides and easily interpretable index of weather the rehabilitation stay is associated to a discrete change in the patient’s disability in activities of daily living. Additionally, the class transition was selected as a measure of a discrete change in the overall disability burden [27, 54], given that the Minimal Clinical Important Difference, MCID, has not been validated yet on the mBI with range 0–100.

## Conclusions

This study focused on the first-step analyses for the development of computational solutions for the clinical decision support. More specifically, a predictive model for functional outcome of post-stroke patients was developed and cross-validated obtaining good accuracies and patient-wise interpretable results of the features contributing to the prediction. This work could be helpful for complementing the assessment of post-stroke patients in the rehabilitation care with evidence-based data and opening the way toward the development of solutions for an optimised and personalised treatment.

## Data Availability

Data is available for research purposes upon request to the corresponding author.

## Abbreviations

CIRS: Cumulative Illness Rating Scale
KNN: K-Nearest Neighbours
SDC: Communication Disability Scale
mRS: Modified Rankin Scale
SAHFE: Standardised Audit of Hip Fracture in Europe
mBI: Modified Barthel Index
RF: Random Forest
SVM: Support Vector Machine
TCT: Trunk Control Test
NRS: Numerical Rating Scale
MMSE: Mini-Mental State Examination
